# Ophthalmic Workplace Modifications for the Post-COVID Era

**DOI:** 10.18502/jovr.v15i3.7458

**Published:** 2020-07-29

**Authors:** Hasan Naveed, Victor Leung, Mehran Zarei-Ghanavati, Christopher Leak, Christopher Liu

**Affiliations:** ^1^Sussex Eye Hospital, Brighton and Sussex University Hospitals NHS Trust, UK; ^2^Brighton and Sussex Medical School, UK; ^3^Core Extension Health & Safety, Canada; ^4^Eye Research Center, Farabi Eye Hospital, Tehran University of Medical Sciences, Tehran, Iran; ^5^Moorfields Eye Hospital, Croydon, UK; ^6^Tongdean Eye Clinic, UK

**Keywords:** COVID-19, Ophthalmic Workplace, Protecting Healthcare Workers, SARS-CoV-2, Personal Protective Equipment

## Abstract

The COVID-19 pandemic necessitates implementation of exposure control measures in all facets of the healthcare sector. Healthcare professionals who work in busy ophthalmology clinics and theaters are amidst the highest at-risk of contracting COVID-19. The authors review the up-to-date scientific evidence of SARS-CoV-2 transmission to demystify and explain the exposure control options available for ophthalmic workplace and offer insights from an industrial hygiene standpoint. As the we enter the post-COVID world, these measures will be critical to enhance workplace safety, and thus protect patients and staff alike.

##  INTRODUCTION 

COVID-19, caused by novel severe acute respiratory syndrome coronavirus 2 (SARS-CoV2), was declared a pandemic by the World Health Organization (WHO) on March 12, 2020.^[1]^ It has since displayed its destructive potential by overwhelming the hospital systems of some of the most well-resourced countries and claiming over 360,000 fatalities in its wake.^[2]^ Furthermore, the cessation of nonurgent elective work in order to increase capacity to manage the predicted surge of cases, whilst necessary, has had significant impact on the care of patients with other comorbidities by restricting access and delaying treatments.

Measures including national lockdowns, enforcing social distancing measures, contact tracing, universal masking, shielding of the vulnerable, and quarantining the infected may have slowed the spread of the disease and reduced impact. In fact, most countries have now passed the peak of their COVID-19 cases. However, without a vaccine there is little evidence to suggest that the complete eradication of COVID-19 will be achieved and experts predict recurrent post-pandemic outbreaks. We have to accept that the virus will be a part of our lives for the foreseeable future.^[3]^

The challenge for healthcare professionals now is to safely resume the necessary healthcare services. The onus is to ensure that this time is used as a valuable opportunity and collective expertise is utilized to innovate genuine improvements for the ultimate benefit of patients.

##  SARS-CoV-2 Description 

SARS-CoV2 is a positive-sense single-stranded RNA virus which infects cells in the lower respiratory tract, gaining entry via the ACE2 receptors, similar to SARS-CoV.^[4]^ Infected individuals can remain asymptomatic or develop symptoms that are predominantly on the respiratory pathological spectrum.^[5,6]^ Most common symptoms experienced are mild in nature and include anosmia, sore throat, cough, fever, and myalgia; however 15% get affected by severe pneumonia leading to acute respiratory distress syndrome requiring intensive care.^[6,7]^ The virus has a propensity to affect the elderly, the comorbid, and the immunocompromised more severely; however, this is not exclusive as an increased viral dose and inoculation can also lead to severe disease phenotype.^[4,8]^

Early analysis by Liu et al (2020) has shown that viral shedding and viral load found in the nasopharyngeal mucosa are directly proportional to the severity of symptoms experienced.^[9]^ It has also been shown that asymptomatic individuals are capable of spreading the virus as well.^[10]^ SARS-CoV2 is highly efficient in its transmission from person to person for a low infective dose, and primarily spreads via respiratory droplets, aerosols, and contact.^[4]^ The total number of particles and ratio of respiratory aerosols (< 5µm) to droplets (> 5µm) is directly proportional to the airway effort and disruption involved in breathing, speaking, coughing, or sneezing.^[11,12,13]^ This is important because larger droplets of respiratory origin do not usually travel more than 2 m, but smaller aerosolized particles can travel further and remain in the air for longer durations.^[11,14]^ For example, particles with aerodynamic diameters of 0.5 and 10 µm will settle 5 feet in 41 h and 8.2 min, respectively.^[14]^ These aforementioned facts make it scientifically plausible that infected individuals constantly spread the virus in their environment, where it lasts in air attached to the aerosols, and also on inanimate surfaces such as plastics for up to 72 h.^[15]^

##  Enhancing Safety in Ophthalmology Settings

Many eye units around the globe had come to a halt during the pandemic. For example, in the UK, the Royal College of Ophthalmologists had recommended the suspension of all non-urgent elective eye operations and postponement of low-risk non-urgent outpatient clinics which lasted approximately 12 weeks.^[16]^ These measures, whilst essential, had a significant impact on the organization and delivery of ophthalmic services. Ophthalmic units now face the challenge of resuming services with a tremendous increase in workload through the added backlog which cannot be ignored.

Ophthalmology is already one of the busiest outpatient specialties in healthcare. Each patient's journey includes several healthcare personnel interacting to undertake routine objective assessments which is often followed by specialized imaging. The clinical consultation can take an average of 8 min and includes a close proximity slit-lamp examination to systematically inspect the eye and its adnexa. During the Wuhan outbreak of COVID-19, nosocomial transmission was reported to be highest in ENT and Ophthalmology.^[17]^ The standard high-volume practice observed in ophthalmic units is therefore very high-risk and cannot be underestimated in subjecting staff and patients to contracting SARS-CoV-2.

Thus, the resumption of effective ophthalmic services mandates several key modifications to safely diagnose and treat patients. Exposure mitigation measures in the Ophthalmology need to be specifically tailored including infection control, engineering control, administrative control, and provision of appropriate protective equipment.

### Infection Control 

•Regular handwashing with soap and running water for 20 sec should be followed by staff. Patients should also gel hands on entry to the unit and before leaving. Upon arrival at home, they should again wash hands

•Clinical interactive surfaces should be cleaned with alcohol-based or bleach-based disinfectants. This is also true for slit-lamp and imaging devices.

•All personnel in the eye department should be instructed never to touch face. This is to ensure that there is protection of all mucous membranes; eyes, nose, and mouth.

•There should be strict non-touch of all surfaces unless unavoidable.

•There should be no handshakes between staff and patients.

### Administrative Control

#### Emergency Clinics

•Emergency eye services should continue to run as a priority to provide timely care to those absolute emergencies where there is a risk of loss of sight.

•All emergency cases should undergo telephone triage by a senior clinician to ascertain the urgency of review and/or provide reassurance. An effective alternative is to utilize video consultations, such as the NHS's Attend Anywhere, which has reduced ophthalmic A&E attendances by 30%. The success of the video consultations has pushed for its development in other ophthalmic specialties.

•It is also recommended that clinicians use a standardized questionnaire during appointment bookings and selection to flag out high-risk patients based on reported symptoms and contact history.

#### Routine Clinics

•Restarting clinical work will require case selection so as to reduce any excessive and avoidable exposure to staff and patients, many of whom are elderly and at an increased risk of becoming infected leading to severe illness with a high mortality rate.

•Stratification systems to prioritize patients will need to be developed on the basis of potential harm and urgency due to delay. Patients who have been postponed and now require urgent treatment will need to have virtual reviews with their notes to classify and prioritize their reviews/treatment.

•Face-to-face time and slots in clinics still need to be minimized. Virtual clinics and telephone triage should be done in specially designated clinics with the patients' notes available. This can be used to identify patients that necessitate further checks, examination, or procedures in person. Such patients can then be booked into designated face-to-face clinics.

•There should be a dedicated patient-accessible eye hospital website detailing information about common ophthalmic conditions and procedures vetted by the ophthalmologists of that institution. There can be supportive video content exploring expectations of different procedures to educate the patient.

•Hospitals must invest in IT infrastructure for safe remote prescribing by liaising with local and regional pharmacies. This will aid repeat prescriptions and allow clinicians to review any continuing treatments, such as for glaucoma patients.

#### Surgical Theatres

•General anesthetic in all cases should be considered only when absolutely necessary due to intubation being considered as an aerosol-generating procedure (AGP). Local anesthetic, if possible, should be explained to patients and considered.

•Careful consideration needs to be given to cases requiring surgical methods involving exploration of the orbit with high-speed drills should be avoided as it generates aerosols.

•Only operating on absolute emergencies where there is a risk of loss of sight during the pandemic and any further waves of infection.

•Patients undergoing elective procedures should self-isolate for two weeks before the surgery and undergo COVID testing (Antigen PCR) 48 h before the surgery.

#### Clinical Pathways

•Patient pathways and flow through the department needs to be carefully considered so that least number of stations are visited for the different tests and required examinations. The pathways should also consider being seen by the least number of professionals possible.

•Face-to-face clinics should operate on specific time slots with well-staggered time intervals. Patients should come alone, or with one relative/carer.

•Patients should be encouraged to arrive at a specific time and wait in car until called in for their appointment. Upon entry, the patients must be screened again for COVID-19 symptoms.

•Self-check in computerized stations should be placed at the entrances where patient can register for their appointment and staff can remotely inquire about COVID-19 symptoms. There can also be remote temperature-checking equipment for the patient to self-check for fever. This can also be used to take a history from the patient.

•Following the check-in, patient should be allowed to enter the department. All patients should cover their mouth and nose with medical masks, cloth masks, or simply with a piece of cloth (unless contraindicated). Upon entry, they should be directed to the clinic room as quickly as possible without any undue delay and waiting.

•Clinic room face-to-face meeting should be primarily examination-based. All examinations must be focused.

•All explanation and consultations after examinations should be done remotely to reduce face-to-face time and discussion in clinics.

•Conversations should also be kept to a minimum and all patients should be instructed to speak little. There should be no talk during the slit-lamp examination.

•Departmental patient numbers should not accrue more than an upper limit, which is defined on the basis of physical space available and ventilation.

#### Staff

•Staff should not accrue in rooms and maintain a distance of at least 1 m at all times.

•There should not be any face-to-face meetings, and where possible telephone and e-meetings should be organized instead.

•Universal masking throughout the premises should be mandated for all staff (unless contraindicated).

### Environmental Control 

#### Patient separation 

•There should be a red pathway for COVID positive, symptomatic, and suspect patients who necessitate examination, with a separate entrance and exit and designated room for examination. Red-labelled operating rooms should also be separated.

•For all patients who are COVID-negative or asymptomatic, there should be a green stream involving a separate entrance and exit. There should also be designated rooms for examination and operating.

#### Outpatients 

•There should be a designated separate entrance and exit for all attending patients.

•Two separate doorways should be individually designated and labelled as exit and entrance for clarity of staff and patients alike.

•There should be flow markings on the floor, and directions on the walls (similar to supermarkets).

•Consider setting up a physical barrier (such as a plexiglass wall) between reception and patient area and adjust ventilation system to have the reception area a positive pressure zone. This way, any direct droplet and airborne particle spread can be blocked from the patient areas.

•Waiting room must have a seating arrangement with a gap of at least 1 m between seats.

#### Clinics and Equipment

•Ophthalmic clinics must be conducted in a designated well-ventilated room. Ventilation systems should be fitted with high efficiency particulate air (HEPA) filters to purify air in the clinical areas. If this is not possible, windows can be opened to maintain airflow in spacious rooms.

•All slit lamps should be modified to incorporate a clear thick plastic or Perspex shield breath guards with sufficient dimensions to provide cover for the examining ophthalmologist and patient.

•In imaging clinics, the machinery can be encompassed by a specialized Perspex box-type enclosure with glove entrances. These enclosures can be linked to the exhaust fan with high efficiency particulate air (HEPA) filter to create negative pressure system for your operation.

#### Operating Theatres 

•Operating theatres should be properly ventilated to meet the designed air change requirement or with ambient air recycled with HEPA systems to reduce risk of aerosol spread and deposition in the environment. ASHRAE recommends 20 air changes per hour for operating rooms.^[18]^

•There should be a designated separate entrance and exit to the theatre.

### Personal Protective Equipment

Personal protective equipment (PPE) provisions are paramount in reducing transmission of SARS-CoV-2 to the healthcare workers. Staff must also be trained in the proper donning and doffing procedures.

#### Eye protection 

Eye protection encompasses several different types of safety devices including safety goggles, visors, and face shields. Ideally, eye protection should be offered in the form of visors or goggles that fit snugly by forming a seal around the eyes. There have been case reports suggesting that coronavirus is transmissible through conjunctival tissue as well.^[19]^

#### Gloves and Gown 

Gloves and gowns are recommended when dealing with high-risk cases, particularly when in an aerosol-generating environment. High-risk areas are cohorted in most hospitals, so the staff should refrain from deploy donning and doffing repeatedly in these areas. PPE donning should only be done in a designated clean area while a separate designated area should be used for PPE removal. This is because doffing can shed virus that may become airborne and contaminate bits of gear, face, or hands. Every surface including used PPE should be considered contaminated. Gloves use should be mandatory to prevent cross-contamination between hand-surface material cross-contamination and when examining ocular and adnexal tissues.

#### Masks and Respirators

Formal use of surgical masks has been recommended at least since the early 1900s to avoid droplet contamination during surgical procedures.^[20]^ Respiratory personal protection is paramount as it protects the mucosa of nose and mouth. Nowadays, there are various types of surgical masks and respirators available to the healthcare workforce (Table 1).^[21,22]^

Surgical masks comprise of three layers: an inner soft absorbent layer, a middle polypropylene barrier, and an outer hydrophobic fabric which mold to the user's nasal bridge to cover nose, mouth, and chin. They act as a barrier to infectious droplets. They also assist in the maintenance of a sterile field by reducing the spread of droplets from the wearer's nose and mouth.^[13,23,24]^

Filtering facepiece respirators (FFPs), on the other hand, provide additional benefit to surgical masks by providing an air-tight seal and containing a mechanical filter, which can remove airborne contaminants through interception. Health and Safety Executive and British Safety Industry Federation recommend fit testing to ensure the respirator is suited to the user's facial structure and therefore performs optimally. There are three categories of FFP in Europe: FFP1, FFP2 (equivalent to N95), and FFP3. Class three (FFP3) provides the highest quality of protection and is the only one approved for UK healthcare settings, especially in AGPs, such as intubation and non-invasive ventilation. They must meet industry-standard regulations including strict industry tests with biological aerosols and cannot exceed 2% leakage. FFP3 masks provide 99% efficiency in filtering particles sized above 100 nm, including small airborne droplets.^[22,24]^

Based on the scientific rationale provided in this article and established practice in the Far-Eastern countries, masks or facial coverings must be worn by all patients and staff at all times.^[25]^ This reduces the spread of respiratory droplets in the environment and deposition on fomites and therefore protects from inhalation of virus-laden aerosols. Close contact with symptomatic patients who have more respiratory aerosol production necessitates the use of respirators.

**Table 1:** Comparison of different commonly used face mask/respirator in the market${}^{\left[21,22\right]}$


**Mask Type**	**Standards**	**Filtration Effectiveness**		
Surgical Mask	China YY 0469	<3.0 microns ≥ 95%		
	<0.1 microns ≥ 30%		
				
	USA ASTM F2100	Level 1	Level 2	Level 3
	3.0 microns ≥ 95%	3.0 microns ≥ 98%	3.0 microns ≥ 98%
	0.1 microns ≥ 95%	0.1 microns ≥ 98%	0.1 microns ≥ 98%
				
	Europe EN 14683	Type I	Type II	Type III
	3.0 microns ≥ 95%	0.3 microns ≥ 98%	0.3 microns ≥ 98%
	0.1 microns **X**	0.1 microns **X**	0.1 microns **X**
Respirator Mask	USA NIOSH (42 CFR 84)	N95/KN95	N99/KN99	N100/KN100
	China GB2626	0.3 microns ≥ 95%	0.3 microns ≥ 99%	0.3 microns ≥ 99.97%
	Europe EN149 :2001	FFP1	FFP2	FFP3
	0.3 microns ≥ 80%	0.3 microns ≥ 94%	0.3 microns ≥ 99%
3.0 microns: Bacteria Filtration standard (BFE) 0.1 microns: Particle Filtration Efficiency standard (PFE) 0.3 microns: Used to represent the most penetrating particle size **X**: No requirements				

##  Conserving and Procuring Personal Protective Equipment

It is envisioned that by streamlining the service during the COVID-19 pandemic to exclusively treat emergencies and operating on the life-, limb-, or organ-threatening conditions, we would conserve the limited supply of specialized protective equipment necessary to provide safe care. However, if this pandemic lasts for a longer duration, with more and more people being infected, then we may be threatening to fast deplete our supply of resources and PPE kit with the current model of single use or single session use.

The issue of ensuring a steady supply of PPE equipment will require commissioning production of these items nationally. In the UK, industrial plants that have machinery to produce face shields or masks have already started to offer a helping hand to the government by developing and supplying PPE to hospitals. Similarly, there are also in-house 3D printing technology centers that can be used to make face shields, visors, and goggles. Countries in short supply need to explore the possibility of further importing from China, where they now have spare capacity. As large variation in quality in masks have been reported, PPE procurement should obtain small batch samples to verify PPE effectiveness prior to mass ordering.

Given the finite resources, it is also paramount that mask reusability and extended wear is explored as a priority. Taiwan was able to successfully limit its public to purchase a maximum of two masks per week, which allowed appropriate distribution to all and enforced reuse.^[26]^ Masks have been shown to be reusable in studies using energetic methods such as germicidal ultraviolet light and microwaved steam.^[27,28,29]^ US Food and Drug Administration has given an emergency go-ahead to a novel Battelle Critical Care Decontamination System${}^{\mathrm{TM}}$ which uses vaporized hydrogen peroxide to sterilize and does not degrade filter performance of respirators.^[30]^ There are also unlicensed recommendations to utilize autoclaves or ovens to sterilize masks and perhaps that will be effective for a limited number of cycles before the mask efficiency degrades to substandard levels. In these desperate times, we have no choice but to be resourceful and adapt during this pandemic by using the existing scientific knowledge of decontamination with our understanding of SARS-CoV-2 virus.^[29]^

##  Conserving Medical, Nursing, and Allied Healthcare Workforce

Healthcare workers are at the most risk of repetitive exposure to this pathogen that increases their viral load predisposing them to contracting severe pneumonia and end up hospitalized. There have several fatalities of frontline staff around the world; in Italy, ∼8% of the total cases affected were healthcare workers.^[31]^ This risk is highest to healthcare workers participating in AGPs and to ENT and Ophthalmology staff who see an increased outpatient load at close quarters.^[17]^

Risk assessment of BAME, co-morbid, and older members of staff should be prioritized to ensure safe working standards and appropriate job roles are allocated. We also have to ensure that staff are tested regularly to maintain a strong battlefront against this pandemic. It should also be compulsory for staff to self-monitor twice daily temperatures and development of infective symptoms, as was done in National University Hospital, Singapore.^[32]^ Depending on the availability of tests, regular SARS-CoV-2 testing should also be mandated for all working and returning staff to prevent asymptomatic spread in the healthcare workplace.^[33]^

##  Summary

*Veni, vidi, vici*. I came, I saw, I conquered. COVID-19 came and unleashed untold harm to society and will linger on with possibility of further waves unless it self-attenuates, or safe effective vaccines arrive. In the light of current knowledge of COVID-19, we have put together important considerations for protecting patients and staff.

*Primum non nocere* – we need to be certain that we are protecting patients from contracting the coronavirus. At the same time, we also have a duty of care to staff. There is not only a contractual duty but a moral duty to protect medical, nursing, and other healthcare staff. Conserving highly trained healthcare staff is of course a national interest.

The prevention of spread is done through education, guidelines, rules, and the law. It behooves governments and healthcare regulators to put these in place. In a century's time, we want future ophthalmologists to be able to look back and say their forebears had done well in the year 2020 protecting ophthalmology patients and staff and laid foundations for new pathways and new ways of delivering safe ophthalmic care, thus transforming ophthalmology despite the challenge of high volume and high risk.

##  Financial Support and Sponsorship

None.

##  Conflicts of Interest

There are no conflicts of interest.
